# *Phocaeicola coprocola* attenuates liver fibrosis by modulating extracellular matrix remodeling

**DOI:** 10.1080/19490976.2026.2718623

**Published:** 2026-08-17

**Authors:** Kyeong Jin Lee, Jeong Ha Park, Hyunjoon Park, Min Ju Kim, Hoyeong Yoon, Jeong Min Kim, Sang Hak Han, Ki Tae Suk

**Affiliations:** a Institute for Liver and Digestive Diseases, Hallym University, Chuncheon, Republic of Korea; b Department of Pathology, Hallym University College of Medicine, Chuncheon, Republic of Korea

**Keywords:** *Phocaeicola coprocola*, gut–liver axis, gut microbiota, extracellular matrix, intestinal barrier

## Abstract

Chronic liver diseases, including metabolic dysfunction–associated steatotic liver disease (MASLD), frequently progress to liver fibrosis, yet effective antifibrotic therapies remain limited. Here, we investigated the therapeutic potential of the commensal bacterium *Phocaeicola coprocola* using Western diet (WD)–induced MASLD and DDC + TAA–induced fibrosis murine models. In the WD model, *P. coprocola* administration attenuated hepatic steatosis, reduced lipogenic gene expression, and improved metabolic and histological parameters. In contrast, in the DDC + TAA model of advanced fibrosis, *P. coprocola* significantly reduced cholestatic markers and fibrosis severity. Mechanistically, these antifibrotic effects occurred independently of broad suppression of inflammatory mediators or upstream TGF-β–Smad signaling, and were instead associated with selective downregulation of extracellular matrix (ECM)–related fibrogenic programs, including *Col1a1*, *Col3a1*, and *Mmp2*. Notably, these effects were observed even in the absence of detectable gut colonization, suggesting that stable engraftment is not required for therapeutic activity. *P. coprocola* also enhanced colonic epithelial barrier–related gene expression and host–microbial metabolic signaling. In humans, circulating ECM remodeling markers (PIIINP, MMP2, and TIMP1) were associated with fibrosis severity, supporting the translational relevance of ECM-targeted mechanisms. Collectively, these findings identify *P. coprocola* as a selective modulator of ECM remodeling that uncouples fibrotic output from upstream inflammatory signaling, highlighting its potential as a microbiome-based therapeutic strategy for liver fibrosis across multiple disease etiologies.

## Graphical Abstract

**Figure:**
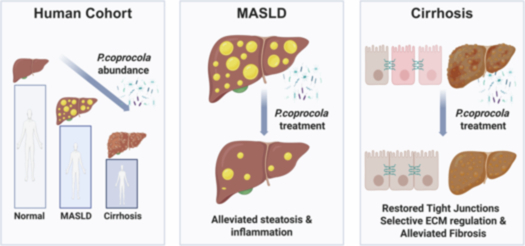

## Introduction

The global burden of metabolic dysfunction–associated steatotic liver disease (MASLD) is rising rapidly, with hepatic fibrosis representing the principal determinant of liver-related morbidity and mortality across the full spectrum of chronic liver disease, including MASLD and advanced cirrhosis.^1^ ^,^ ^2^

Fibrosis results from excessive accumulation of extracellular matrix (ECM), produced predominantly by activated hepatic stellate cells (HSCs)^3^ ^,^ ^4^ In response to chronic liver injury—whether driven by metabolic dysfunction, cholestatic insult, or toxic stimuli—quiescent HSCs transdifferentiate into *α*-smooth muscle actin–expressing myofibroblast-like cells^5^ and secrete fibrogenic collagens, including types I and III.^6^ Because fibrosis stage predicts clinical outcomes more strongly than isolated steatosis,^7^ ^,^ ^8^ therapeutic strategies that interrupt ECM production or disrupt pathways sustaining HSC activation are urgently needed.^9‐11^

Increasing evidence implicates the gut–liver axis as a modifiable driver of MASLD progression and fibrogenesis.^12^ ^,^ ^13^ Gut microbiota dysbiosis and impaired intestinal barrier integrity promote the translocation of microbial products^14^ ^,^ ^15^ and metabolites, amplifying hepatic inflammation, metabolic dysfunction, and stellate-cell activation, thereby accelerating ECM deposition. Accordingly, strategies that restore microbial homeostasis or reinforce mucosal barrier function represent promising approaches to mitigate gut-to-liver fibrogenic signaling and slow disease progression.^16^

Host-adapted commensal microbes with specialized metabolic functions are particularly attractive candidates for precision microbiome therapeutics,^17^ as they can stably occupy ecological niches^18^ and exert sustained effects on host metabolism and immune tone.^19^ ^,^ ^20^

*Phocaeicola coprocola(P. coprocola)* is a prevalent human gut commensal and a dominant taxon in the gut microbiome of koalas (*Phascolarctos cinereus*), an herbivorous mammal uniquely adapted to a lipid- and fiber-rich, toxin-laden eucalyptus diet.^21^ Functional analyzes of the koala microbiome reveal enrichment of pathways involved in lipid and complex carbohydrate metabolism,^21^ suggesting that *P. coprocola* possesses inherent metabolic versatility.

To gain an in-depth understanding of the relationship between gut microbiota and liver disease, we established murine models and performed 16S rRNA sequencing analyzes across multiple disease stages. Integrated multi-omics analyzes identified *P. coprocola* as a microbial taxon whose relative abundance was significantly reduced with MASLD progression, suggesting a potential inverse association with disease severity.

Subsequent functional validation using mouse models demonstrated that *P. coprocola* exerts protective effects against both MASLD and liver fibrosis. Our findings support a model in which *P. coprocola*, as a host-adapted metabolic commensal, may contribute to improved intestinal barrier function and altered gut microbial community structure, accompanied by attenuation of ECM-associated transcriptional programs during liver fibrogenesis.

Here, we investigate the therapeutic potential of *P*. *coprocola* as a gut-derived modulator of ECM remodeling and explore its utility as a microbiome-based strategy for liver fibrosis across distinct disease etiologies.

## Result

### The relative abundance of Phocaeicola coprocola progressively decreases with liver disease severity

**Figure 1.**
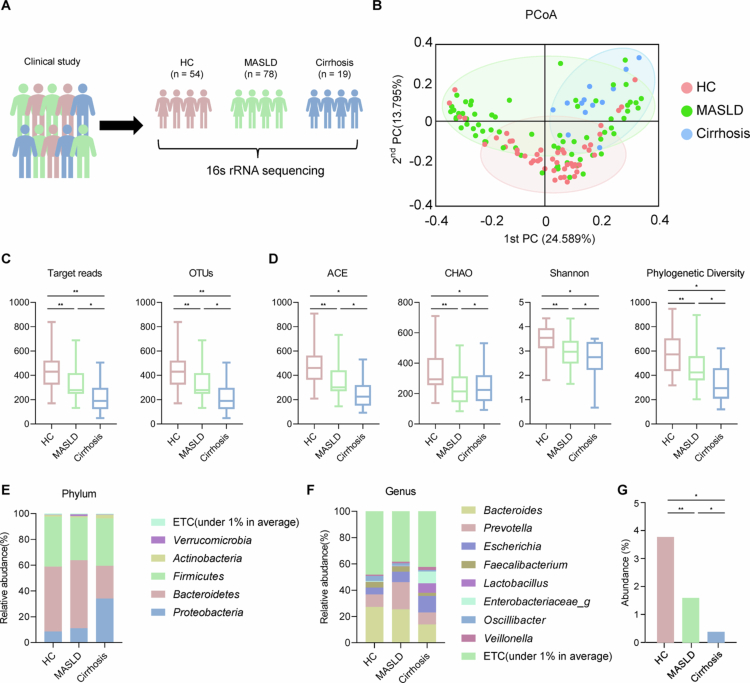
The relative abundance of *Phocaeicola coprocola* progressively decreases with liver disease severity. (A) Study design and subject classification for 16S rRNA gene sequencing analysis (HC, *n* = 54; MASLD, *n* = 78; Cirrhosis, *n* = 19). (B) Principal coordinate analysis (PCoA) plot showing distinct clustering of gut microbial communities based on Jensen-Shannon distance. (C) Sequencing depth (target reads) and number of observed OTUs across groups. (D) Alpha diversity indices (ACE, Chao1, Shannon, and phylogenetic diversity) showing progressive decline in MASLD and cirrhosis. (E) Relative abundance of gut microbiota at the phylum level in each group. (F) Genus-level taxonomic composition, highlighting shifts in beneficial and potentially pathogenic taxa. (G) Relative abundance of *P. coprocola* across groups. Data in (C), (D), and (G) are presented as box-and-whisker plots (median, interquartile range, and min–max) or relative abundance (%), with statistical significance determined using the Kruskal-Wallis test followed by Dunn's multiple comparisons test. *P < 0.05, **P < 0.01. Abbreviations: OTUs, Operational Taxonomic Units; ACE, Abundance-based Coverage Estimator; PCoA, Principal Coordinate Analysis.

To characterize the structural and specific alterations in the gut microbiota associated with MASLD progression, we analyzed a clinical cohort comprising 151 participants: healthy controls (HC, *n* = 54), patients with MASLD (*n* = 78), and patients with liver cirrhosis (*n* = 19) (Figure 1A). Principal coordinate analysis (PCoA) based on beta-diversity metrics revealed a distinct separation of microbial communities among the three groups. Notably, the microbiome of patients with cirrhosis formed a cluster divergent from that of HCs, indicating a profound reconfiguration of the gut ecosystem correlated with disease severity (Figure 1B).

While sequencing depth, as reflected by target read counts, was comparable across groups (Figure 1C), *α*-diversity analyzes revealed a pronounced erosion of microbial richness and evenness with advancing disease. The number of observed operational taxonomic units (OTUs) was markedly reduced in patients with cirrhosis compared with healthy controls (HCs) and patients with MASLD. Consistently, indices reflecting community richness (ACE and Chao1) and overall diversity (Shannon and phylogenetic diversity) showed a stepwise decline from health to MASLD and cirrhosis, indicating that liver disease progression is associated with a progressive loss of gut microbial complexity (Figure 1D).

Taxonomic profiling further elucidated these ecological shifts. At the phylum level, although Firmicutes and Bacteroidetes remained dominant across all groups, disease progression was associated with an expansion of Proteobacteria—a phylum enriched in potential pathobionts—accompanied by a concomitant reduction in Bacteroidetes (Figure 1E). At the genus level, this dysbiotic pattern was marked by depletion of health-associated taxa, including *Faecalibacterium* and *Bacteroides*, together with enrichment of opportunistic genera such as *Escherichia* and unclassified *Enterobacteriaceae* in patients with cirrhosis (Figure 1F).

Crucially, to identify microbial taxa with potential therapeutic relevance, we examined species-level abundance profiles. *Phocaeicola coprocola*(*P. coprocola*) emerged as a key taxon that was progressively depleted with disease advancement. The relative abundance of *P. coprocola* was significantly reduced in patients with MASLD compared with healthy controls and was nearly undetectable in patients with cirrhosis (Figure 1G). This profound and progressive depletion suggests that the loss of *P. coprocola* may be a contributing factor to, or a consequence of, the loss of gut homeostasis in chronic liver disease.

### Phocaeicola coprocola alleviates Western diet–induced hepatic steatosis and liver injury

**Figure 2.**
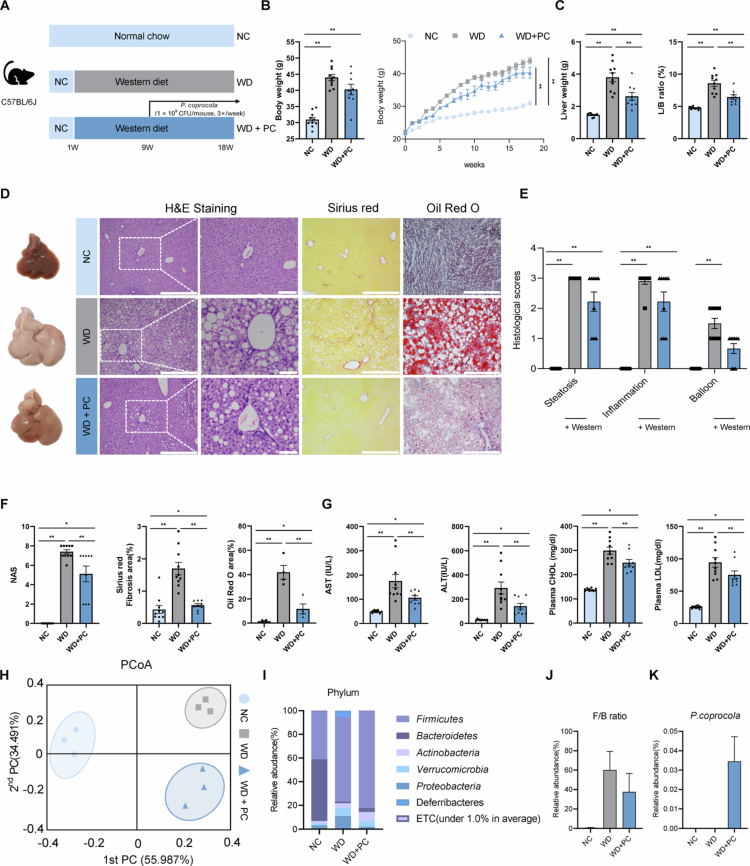
Phocaeicola coprocola alleviates Western diet–induced hepatic steatosis and liver injury. (A) Experimental design of the animal study. (B) Body weight at the final time point and longitudinal body weight changes across groups. Longitudinal body weight trajectories were analyzed using a two-way repeated-measures analysis (mixed-effects model with REML) (Group *P* < 0.0001, Time *P* < 0.0001, Group × Time interaction *P* < 0.0001). Pairwise comparisons at week 18 were performed using one-way ANOVA followed by Tukey's multiple comparisons test. (C) Liver weight and liver-to-body weight ratio across groups. (D) Representative images of liver sections stained with H&E, Sirius Red, and Oil Red O. All images were acquired at 100 × magnification (scale bars, 500 μm), except for the H&E images on the right, which represent 2 × digital magnification of the indicated regions (scale bar, 250 μm). (E) Individual histological scores. (F) Quantitative analysis of liver histology. (Left) NAFLD activity score (NAS). (Middle) Sirius Red–positive fibrotic area. (Right) Oil Red O (ORO)–positive lipid area. (G) Serum biochemical parameters, including AST, ALT, total cholesterol, and LDL. (H) Principal coordinate analysis (PCoA) of gut microbiota based on Bray–Curtis dissimilarity. (I) Relative abundance of gut microbiota at the phylum level. (J) Firmicutes/Bacteroidetes (F/B) ratio. (K) Relative abundance of *P. coprocola*. Data are presented as mean ± SEM unless otherwise stated. Histological scores (E) are presented as mean ± SD. Statistical significance was determined by ordinary one-way ANOVA followed by Tukey's multiple comparisons test, except for the longitudinal body weight analysis in (B), which was analyzed using a two-way repeated-measures analysis (mixed-effects model with REML), and the histological scores (E), which were analyzed using the Kruskal–Wallis test followed by Dunn's multiple comparisons test. *P* < 0.05, **P < 0.01. Abbreviations: AST, Aspartate Aminotransferase; ALT, Alanine Aminotransferase; LDL, Low-Density Lipoprotein; NAS, NAFLD Activity Score.

To elucidate the direct effects and underlying mechanisms of *P. coprocola*, we performed rigorous preclinical experiments using a well-established murine model of metabolic dysfunction–associated steatotic liver disease (MASLD) (Figure 2). Male C57BL/6J mice were randomly assigned to three groups (*n* = 10 per group): a normal chow (NC) diet group, a Western diet (WD; 42% kcal from fat, primarily derived from milk fat and lard; 42.7% carbohydrate and 15% protein) group, and a WD group supplemented with oral *P. coprocola* (1 × 10^9^ CFU per mouse). Mice were fed the assigned diets for 18 weeks, and *P. coprocola* was administered orally three times per week beginning at week 9 of WD feeding until the end of the study (WD + PC) (Figure 2A). Body and liver weights were measured at the end of the experiment. WD feeding resulted in a marked increase in body weight, liver weight, and the liver-to-body weight ratio (L/B ratio) compared with NC-fed controls. In contrast, *P. coprocola* supplementation significantly attenuated liver enlargement and reduced the L/B ratio, while body weight remained comparable to that of WD-fed mice (Figure 2B and 2C).

Histological examination revealed that WD feeding induced pronounced hepatic steatosis, hepatocellular ballooning, and inflammatory infiltration, as assessed by hematoxylin and eosin (H&E) staining. These pathological features were substantially ameliorated in mice receiving *P. coprocola*, indicating a marked protective effect against diet-induced liver injur These pathological features were substantially ameliorated in mice receiving *P. coprocola*, supporting its protective effects against diet-induced liver injury (Figure 2D). Consistently, Oil Red O (ORO) staining demonstrated extensive lipid accumulation in the livers of WD-fed mice, whereas *P. coprocola* supplementation markedly reduced intrahepatic lipid deposition. Moreover, Sirius Red staining revealed increased collagen deposition in the livers of WD–fed mice, which was markedly reduced following *P. coprocola* treatment, indicating an attenuation of hepatic fibrosis. Quantitative histological scoring further confirmed significant reductions in steatosis, inflammation, and hepatocellular ballooning, leading to a decreased NAFLD activity score (NAS) in *P. coprocola*–treated mice (Figure 2D-F). In line with these histological improvements, serum levels of aspartate aminotransferase (AST) and alanine aminotransferase (ALT) were significantly reduced by *P. coprocola* supplementation. In addition, plasma total cholesterol and low-density lipoprotein (LDL) levels were markedly decreased, reflecting an overall improvement in WD-induced metabolic and hepatic injury (Figure 2G).

### P. coprocola partially reshapes gut microbial composition under Western diet conditions

To investigate whether the hepatoprotective effects of *P. coprocola* were associated with alterations in gut microbiota composition, we performed 16S rRNA gene sequencing on fecal samples. Principal coordinate analysis (PCoA) revealed distinct clustering patterns among the three groups, with the WD + PC group showing a microbiota profile intermediate between NC and WD groups (Figure 2H). Alpha diversity indices, including ACE, Chao1, and Shannon index, demonstrated that WD feeding reduced microbial diversity, and *P. coprocola* supplementation partially recovered this diversity loss (Figure 2A, left panels). At the phylum level, the WD group showed altered proportions of major phyla including Firmicutes and Bacteroidetes compared to NC, while *P. coprocola* treatment modulated these alterations (Figure 2I). Phylogenetic diversity analysis at both phylum and family levels further supported improved microbial community richness following *P. coprocola* treatment (Figure 2J, K). At the family level, *P. coprocola* administration was associated with changes in the relative abundance of multiple bacterial families, including increases in Lactobacillaceae and Akkermansiaceae, and decreases in families such as Peptostreptococcaceae and Desulfovibrionaceae (Supplementary Fig. 2 C).

To evaluate the impact of *Phocaeicola coprocola* supplementation on gut microbial composition, fecal samples were collected from mice fed a normal diet (ND), Western diet (WD), or Western diet supplemented with *P. coprocola* (WD + PC), and culturomics analysis was performed. Fecal-derived bacteria were isolated and cultured on modified Gifu anaerobic medium (mGAM) agar without additional supplements, followed by taxonomic identification using matrix-assisted laser desorption/ionization–time of flight mass spectrometry (MALDI–TOF MS) (Supplementary Fig. 2 A).

Venn diagram analysis revealed that five bacterial species were commonly detected across all three dietary groups, while each group also exhibited distinct microbial signatures, indicating diet- and treatment-specific differences in culturable bacterial composition (Supplementary Fig. 1B). Quantitative analysis of isolate frequency further identified significant group-dependent differences in two *Enterococcus* species (Supplementary Fig. 2 C, D). Specifically, *Enterococcus faecalis* was relatively abundant in the ND and WD + PC groups but markedly reduced in the WD group. In contrast, *Enterococcus faecium* was significantly enriched in the WD group, whereas its abundance was reduced in both the ND and WD + PC groups.

To further explore whether *P.coprocola* supplementation induced strain-level differences among gut bacterial isolates, representative *Enterococcus faecalis* strains from each group were subjected to Fourier transform infrared (FT-IR) spectroscopy. Principal component analysis (PCA) of FT-IR spectra revealed that isolates from the WD + PC group exhibited a clustering pattern distinct from that of the WD group, indicating diet- and treatment-associated differences in strain-level modulation (Supplementary Fig. 2E). Collectively, these data indicate that *P. coprocola* supplementation is associated with selective modulation of culturable gut bacterial taxa under WD conditions.

### Phocaeicola coprocola attenuates WD-induced hepatic transcriptional programs governing lipid metabolism, inflammation, and fibrosis

Given that *P. coprocola* supplementation ameliorated WD-induced hepatic injury and modulated gut microbial composition (Figure 2H-K and Supplementary Fig. 2), we next examined whether these changes were associated with alterations in hepatic transcriptional programs related to lipid metabolism, inflammation, and fibrosis. Hepatic gene expression was analyzed by quantitative real-time PCR (qPCR) to assess pathways related to lipid synthesis, lipid uptake, inflammatory responses, and extracellular matrix (ECM) remodeling.

**Figure 3.**
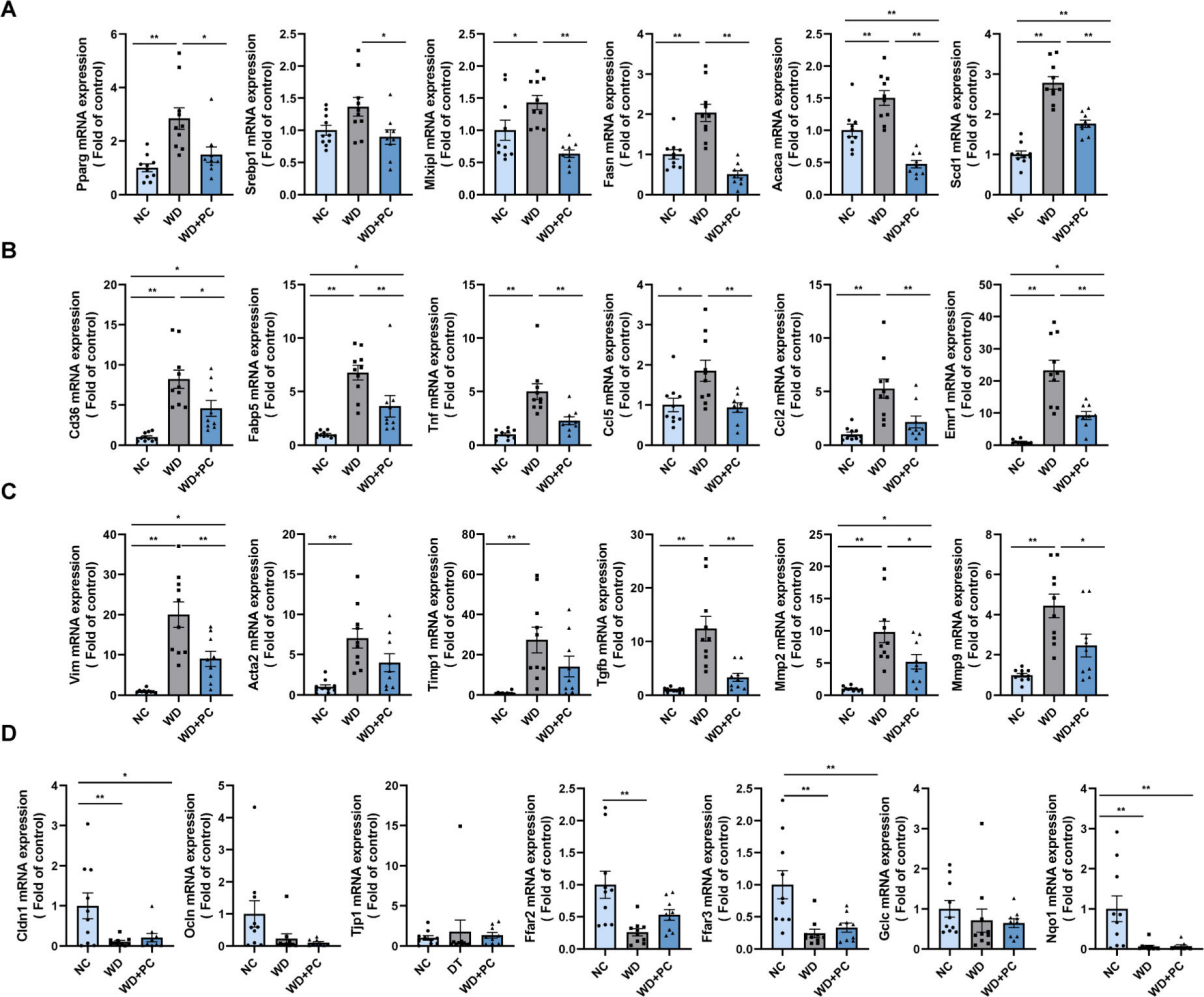
*Phocaeicola coprocola* attenuates WD-induced hepatic transcriptional programs governing lipid metabolism, inflammation, and fibrosis. (A) Hepatic mRNA expression of lipogenesis-related transcription factors and enzymes measured by qPCR (*Pparg*, *Srebp1c*, *Mlxipl*, *Fasn*, *Acaca*, *Scd1*). (B) Hepatic mRNA expression of genes involved in lipid uptake and inflammation measured by qPCR (*Cd36, Fabp5, Tnf, Ccl5, Ccl2, Emr1*). (C) Hepatic mRNA expression of fibrosis- and ECM remodeling–related genes measured by qPCR (*Vim*, *Acta2*, *Timp1*, *Tgfb*, *Mmp2*, *Mmp9*). (D) Colonic mRNA expression of SCFA receptor– and antioxidant response–related genes measured by qPCR (Ffar2, Ffar3, Gclc, Nqo1). Data are presented as mean ± SEM. Statistical significance was determined by ordinary one-way ANOVA followed by Tukey’s multiple comparisons test. *P < 0.05, **P < 0.01. Abbreviations: qPCR, quantitative polymerase chain reaction; ECM, extracellular matrix.

WD feeding markedly activated de novo lipogenesis (DNL)–associated transcriptional programs. Key transcription factors regulating hepatic lipid synthesis, including peroxisome proliferator–activated receptor gamma (*Pparg*), sterol regulatory element–binding protein 1c (*Srebf1*), and carbohydrate-responsive element–binding protein (*Mlxipl*), were significantly upregulated in WD-fed mice. In parallel, downstream lipogenic enzymes involved in fatty acid synthesis, including fatty acid synthase (*Fasn*), acetyl-CoA carboxylase 1 (*Acaca*), and stearoyl-CoA desaturase 1 (*Scd1*), were also significantly increased. Importantly, oral administration of *P. coprocola* significantly attenuated the expression of these transcription factors and enzymes, indicating suppression of WD-induced hepatic lipogenic transcriptional activity (Figure 3A). Consistent with reduced lipid synthesis, genes associated with hepatic lipid uptake and inflammatory responses were also modulated by *P. coprocola*. WD feeding significantly increased the expression of cluster of differentiation 36 (*Cd36*) and fatty acid–binding protein 5 (*Fabp5*), which facilitate fatty acid uptake into hepatocytes. In addition, pro-inflammatory mediators, including tumor necrosis factor-*α* (*Tnf*), C–C motif chemokine ligand 5 (*Ccl5*), monocyte chemoattractant protein-1 (*Ccl2*), and the macrophage-associated marker F4/80 (*Emr1*), were markedly elevated. These increases were significantly attenuated by *P. coprocola* supplementation (Figure 3B).

We next examined genes involved in fibrosis and ECM remodeling. WD feeding significantly upregulated mesenchymal and profibrotic markers, including vimentin (*Vim*), alpha-smooth muscle actin (*Acta2*), tissue inhibitor of metalloproteinases-1 (*Timp1*), and transforming growth factor-β1 (*Tgfb1*). In addition, the expression of matrix remodeling enzymes matrix metalloproteinase-2 (*Mmp2*) and matrix metalloproteinase-9 (*Mmp9*) was significantly induced. Consistent with the reduction in collagen deposition observed by Sirius Red staining (Figure 2C), the expression of these fibrosis- and ECM-related genes was markedly reduced in the *P. coprocola*–treated group compared with WD controls (Figure 3C).

Collectively, these data indicate that *P. coprocola* supplementation is associated with coordinated suppression of WD-induced hepatic transcriptional programs involved in lipogenesis, lipid uptake, inflammatory signaling, and fibrotic remodeling.

### Phocaeicola coprocola supplementation does not induce pathological changes in healthy mice

**Figure 4.**
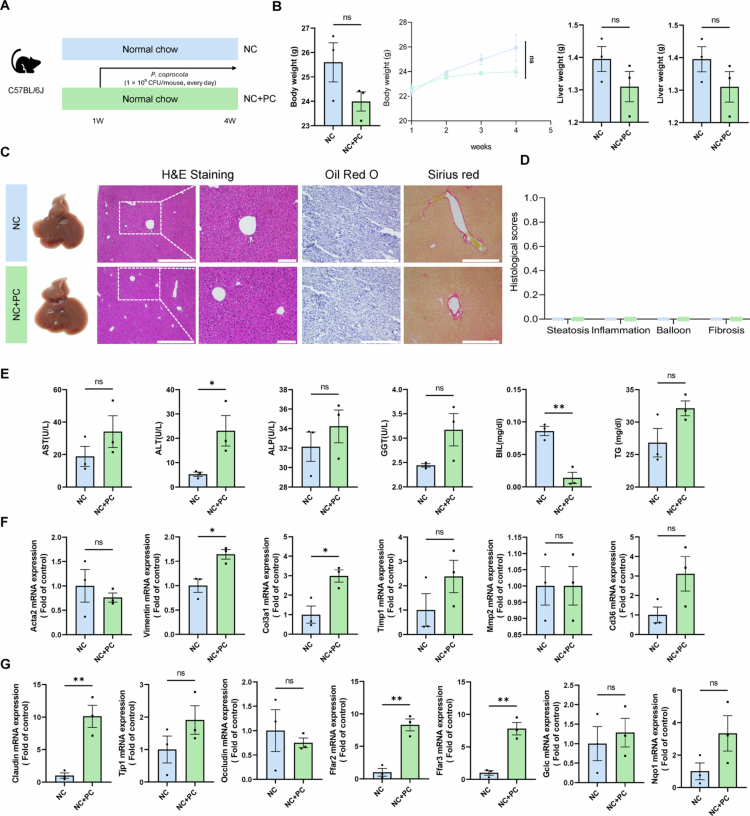
*Phocaeicola* coprocola supplementation does not induce pathological changes in healthy mice. (A) Experimental design of the animal study. (B) Body weight, liver weight, and liver-to-body weight ratio across groups. (C) Representative images of liver sections stained with H&E, Sirius Red, and Oil Red O. All images were acquired at 100 × magnification (scale bars, 500 μm), except for the magnified H&E images on the right, which represent 2 × digital zoom of the indicated regions (scale bar, 250 μm). (D) Individual histological scores. (E) Serum biochemical parameters, including AST, ALT, ALP, GGT, BIL and TG. (F) Hepatic mRNA expression of genes related to fibrosis and ECM remodeling measured by qPCR (Acta2, Vim, Col3a1, Timp1, Mmp2 and Cd36). (G) Colonic mRNA expression of SCFA receptor– and antioxidant response–related genes measured by qPCR (Ffar2, Ffar3, Gclc, Nqo1). Data are presented as mean ± SEM. Statistical significance was determined by unpaired two-tailed Student’s t-test. *P < 0.05, **P < 0.01. Abbreviations: AST, aspartate aminotransferase; ALT, alanine aminotransferase; ALP, alkaline phosphatase; GGT, gamma-glutamyl transferase; BIL, bilirubin; ALB, albumin; TG, triglycerides; ECM, extracellular matrix.

To evaluate the safety profile of *P. coprocola* in the absence of disease, we performed preclinical experiments in healthy mice fed a normal chow diet (Figure 4). Male C57BL/6J mice were randomly assigned to two groups (*n* = 3 per group): a NC diet group and an NC group supplemented with oral *P. coprocola* (1 × 10^9^ CFU per mouse). Mice were fed a normal chow diet for 3 weeks, and *P. coprocola* was administered orally daily throughout the experimental period (NC + PC) (Figure 4A). Body and liver weights were measured at the end of the experiment. Body weight and liver weight remained comparable between NC and NC + PC groups, indicating that *P. coprocola* supplementation does not affect normal growth or hepatic mass in healthy animals (Figure 4B).

Histological analyzes, including H&E, Oil Red O (ORO), and Sirius Red staining, revealed no evidence of steatosis, inflammation, hepatocellular ballooning, or fibrosis in either group (Figure 4C). Quantitative scoring further confirmed the absence of pathological alterations, with all parameters—steatosis, inflammation, hepatocellular ballooning, and fibrosis—scoring zero in both NC and NC + PC groups (Figure 4D).

Serum biochemical analysis showed that most liver function parameters remained within normal ranges. Although ALT was modestly elevated and total bilirubin was slightly reduced in NC + PC mice compared to NC controls, both values remained within physiological limits. Other markers of liver injury, including AST, alkaline phosphatase (ALP), and gamma-glutamyl transferase (GGT), as well as serum albumin and triglyceride levels, were unchanged between groups (Figure 4D). Notably, the absence of histological abnormalities despite the mild ALT elevation suggests that these changes reflect physiological variation rather than hepatotoxicity.

Consistent with the absence of histological and biochemical abnormalities, hepatic expression of fibrosis- and extracellular matrix (ECM)–related genes remained largely unchanged in ND + PC mice compared with NC controls. Although modest but statistically significant increases in *Vim*, *Col3a1*, and *Cd36* were observed, these changes occurred in the absence of histopathological fibrosis and were not accompanied by coordinated upregulation of key fibrogenic markers (*Acta2*, *Timp1*, *Mmp2*). Importantly, expression levels of these genes remained substantially lower than those observed in disease models, and no collagen deposition or architectural disruption was detected histologically (Figure 4A–B). Together, these findings indicate that *P. coprocola* administration does not induce hepatic fibrogenic responses under physiological conditions (Figure 4F). In contrast, colonic gene expression analysis revealed a significant upregulation of epithelial barrier–related genes, particularly *Cldn1*, along with marked increases in SCFA receptors (*Ffar2* and *Ffar3*) in ND + PC mice (Figure 4G). Although changes in antioxidant genes (*Gclc* and *Nqo1*) did not reach statistical significance, they exhibited an increasing trend. These findings suggest that *P. coprocola* enhances colonic barrier function and host–microbial metabolic responsiveness without inducing hepatic alterations.

### Phocaeicola coprocola attenuates DDC + TAA–induced liver fibrosis and injury

**Figure 5.**
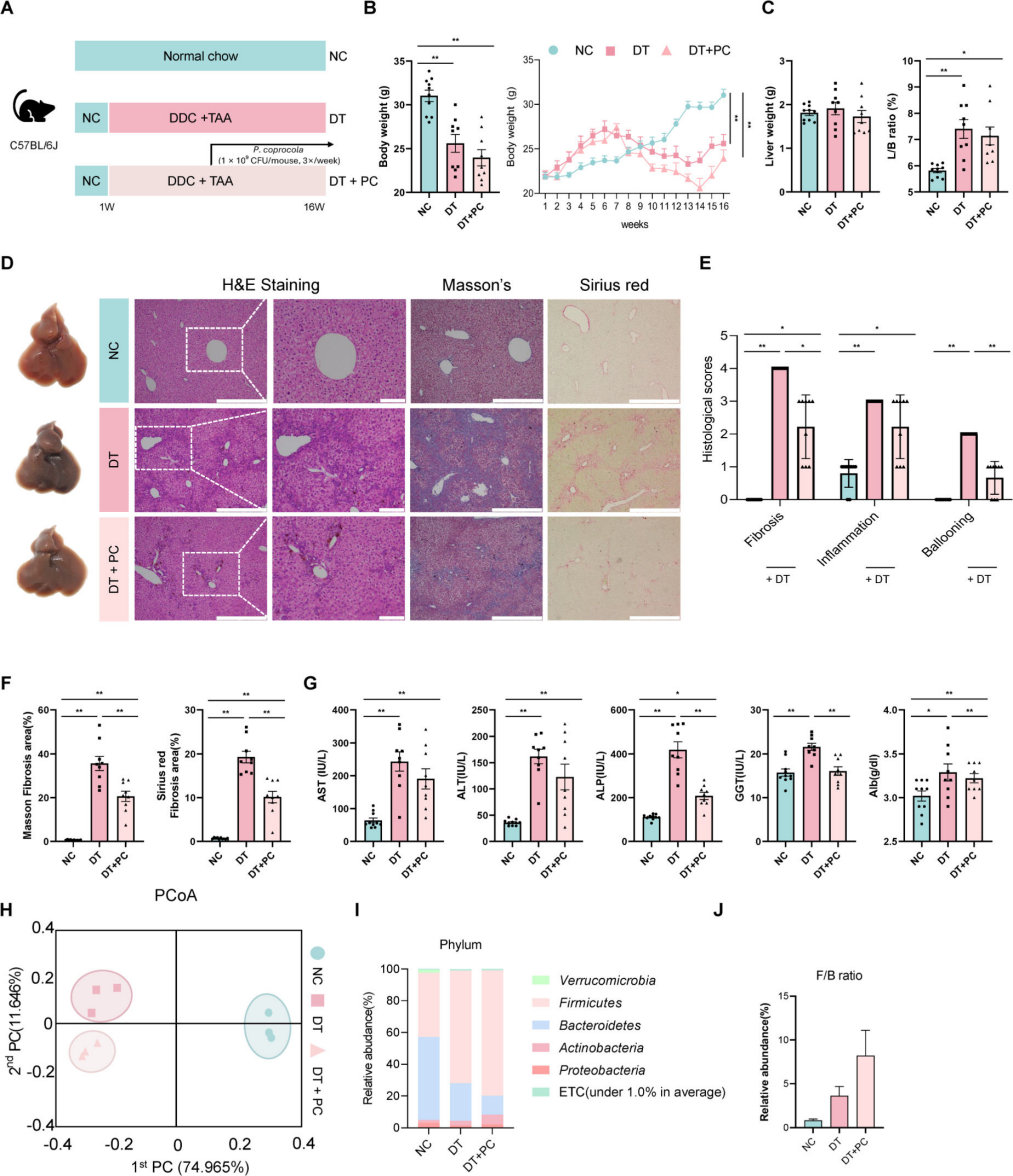
*Phocaeicola coprocola* attenuates DDC + TAA–induced liver fibrosis and injury. (A) Experimental design of the animal study. (B) Body weight at the final timepoint and longitudinal body weight changes across groups. Statistical significance of longitudinal body weight trajectories was determined by two-way repeated measures analysis (mixed-effects model, REML); Group *p* < 0.0001, Time *p* < 0.0001, Group × Time interaction *p* < 0.0001. Pairwise comparisons at week 16 were determined by one-way ANOVA with Tukey's multiple comparisons test. (C) Liver weight and liver-to-body weight ratio across groups. (D) Representative images of liver sections stained with H&E, Masson's trichrome, and Sirius Red. All images were acquired at 100 × magnification (scale bars, 500 μm), except for the magnified H&E images on the right, which represent 2 × digital zoom of the indicated regions (scale bar, 250 μm). (E) Individual histological scores. (F) Quantitative analysis of liver histology. (Left) Percentage of Masson's trichrome-stained fibrosis area. (Right) Percentage of Sirius Red-stained fibrosis area. (G) Serum biochemical parameters, including AST, ALT, ALP, GGT, and albumin (Alb). (H) Principal coordinate analysis (PCoA) plot of gut microbiota based on Bray–Curtis dissimilarity. (I) Relative abundance of gut microbiota at the phylum level. (J) The ratio of Firmicutes to Bacteroidetes (F/B ratio) across groups. Data are presented as mean ± SEM unless otherwise stated. Histological scores (E) are presented as mean ± SD. Statistical significance was determined by ordinary one-way ANOVA followed by Tukey's multiple comparisons test, except for the longitudinal body weight analysis in (B), which was analyzed using a two-way repeated-measures analysis (mixed-effects model with REML), and the histological scores (E), which were analyzed using the Kruskal–Wallis test followed by Dunn's multiple comparisons test. *P < 0.05, **P < 0.01, ***P < 0.001. Abbreviations: AST, aspartate aminotransferase; ALT, alanine aminotransferase; Alb, albumin; NAS, NAFLD activity score.

Given the demonstrated safety profile of *P. coprocola* in healthy mice and its therapeutic efficacy in ameliorating Western diet–induced hepatic steatosis and early fibrotic changes, we next sought to determine whether *P. coprocola* could exert antifibrotic effects in a more clinically relevant model of advanced liver fibrosis. To address this, we employed a combined DDC + TAA model, which induces cholestatic injury and progressive fibroinflammatory liver disease that more closely resembles human cirrhosis. To evaluate the therapeutic potential of *P. coprocola* in a model of advanced fibroinflammatory liver injury, we performed additional preclinical experiments using combined 3,5-diethoxycarbonyl-1,4-dihydrocollidine (DDC) and thioacetamide (TAA). Male C57BL/6J mice were randomly assigned to three groups: NC, DDC + TAA (DT), and DDC + TAA + *P. coprocola* (DT + PC). Mice in the DT and DT + PC groups were fed a diet containing 0.05% DDC and received TAA (200 mg/L) in the drinking water for 10 weeks to induce cholestatic injury and progressive fibrosis. *P. coprocola* (1 × 10^9^ CFU per mouse) was administered orally three times per week starting at the onset of DDC + TAA treatment and continuing throughout the experimental period (Figure 5A). Body and liver weights were measured at the study endpoint. DT treatment led to a significant reduction in body weight compared with NC controls, while absolute liver weight remained comparable among groups. Consequently, the liver-to-body weight (L/B) ratio was significantly increased in DT mice. Notably, while DT + PC mice exhibited modest body weight reduction compared to DT controls throughout the experimental period (Figure 5B), likely reflecting altered metabolic responses under toxin-induced stress, *P. coprocola* supplementation did not significantly affect liver weight (Figure 5C), indicating that its effects were not attributable to changes in liver mass or hepatomegaly.

Macroscopic examination revealed that DT administration induced marked hepatomegaly with visible surface nodularity and discoloration, characteristic of severe fibrotic liver injury. In contrast, livers from DT + PC mice displayed improved gross morphology with reduced surface irregularity (Figure 5D). H&E staining revealed that DT administration induced profound hepatocellular ballooning and inflammatory cell infiltration. Masson's trichrome and Sirius Red staining demonstrated extensive collagen deposition and fibrotic remodeling in the livers of DT mice (Figure 5D, F). These pathological features were substantially ameliorated in mice receiving *P. coprocola*, indicating a marked protective effect against fibroinflammatory liver injury. Quantitative histological scoring further confirmed significant reductions in fibrosis, inflammation, and hepatocellular ballooning in *P. coprocola*–treated mice (Figure 4E). Consistent with these histological improvements, serum levels of liver injury markers including ALT, AST, ALP, and GGT were significantly elevated in DT-treated mice, reflecting severe hepatocellular damage and cholestatic injury. *P. coprocola* supplementation markedly reduced these biochemical markers, indicating restoration of hepatic function (Figure 5G). Furthermore, serum albumin levels, which were significantly decreased in DT-treated mice indicating impaired hepatic synthetic function, were partially restored by *P. coprocola* treatment.

To investigate whether the hepatoprotective effects of *P. coprocola* were associated with alterations in gut microbiota composition, we performed 16S rRNA gene sequencing on fecal samples. Principal coordinate analysis (PCoA) revealed distinct clustering patterns among the three groups, with the DT + PC group showing a microbiota profile that partially diverged from both NC and DT groups (Figure 5H). Alpha diversity indices, including ACE, Chao1, and Shannon index, showed that DDC + TAA treatment did not significantly alter overall microbial diversity, and *P. coprocola* supplementation exhibited modest trends in these metrics (Supplementary Figure 3 A). At the phylum level, the DT group showed increased Firmicutes and decreased Bacteroidetes compared to NC, while *P. coprocola* treatment further modulated these alterations (Figure 4I, Supplementary Figure 3B). At the family level, *P. coprocola* administration was associated with changes in the relative abundance of multiple bacterial families, including *Prevotellaceae, Bacteroidaceae*, *Rikenellaceae*, *Muribaculaceae*, *Coriobacteriaceae*, *Clostridiaceae*, *Lachnospiraceae*, *Erysipelotrichaceae*, *Ruminococcaceae*, *Lactobacillaceae*, *AC160630f*, *Bifidobacteriaceae*, *Desulfovibrionaceae*, *Christensenellaceae*, *Akkermansiaceae*, and *Helicobacteraceae* (Supplementary Figure 3 C). The Firmicutes-to-Bacteroidetes (F/B) ratio was elevated in the DT group and further increased in the DT + PC group (Figure 5J).

Collectively, these data demonstrate that *P. coprocola* exerts potent antifibrotic and hepatoprotective effects in the DDC + TAA model, attenuating histological fibrosis, inflammation, and hepatocellular injury while restoring liver function and modulating gut microbiota composition.

### Transcriptomic analysis identifies extracellular matrix remodeling as a major altered pathway in advanced liver injury

**Figure6.**
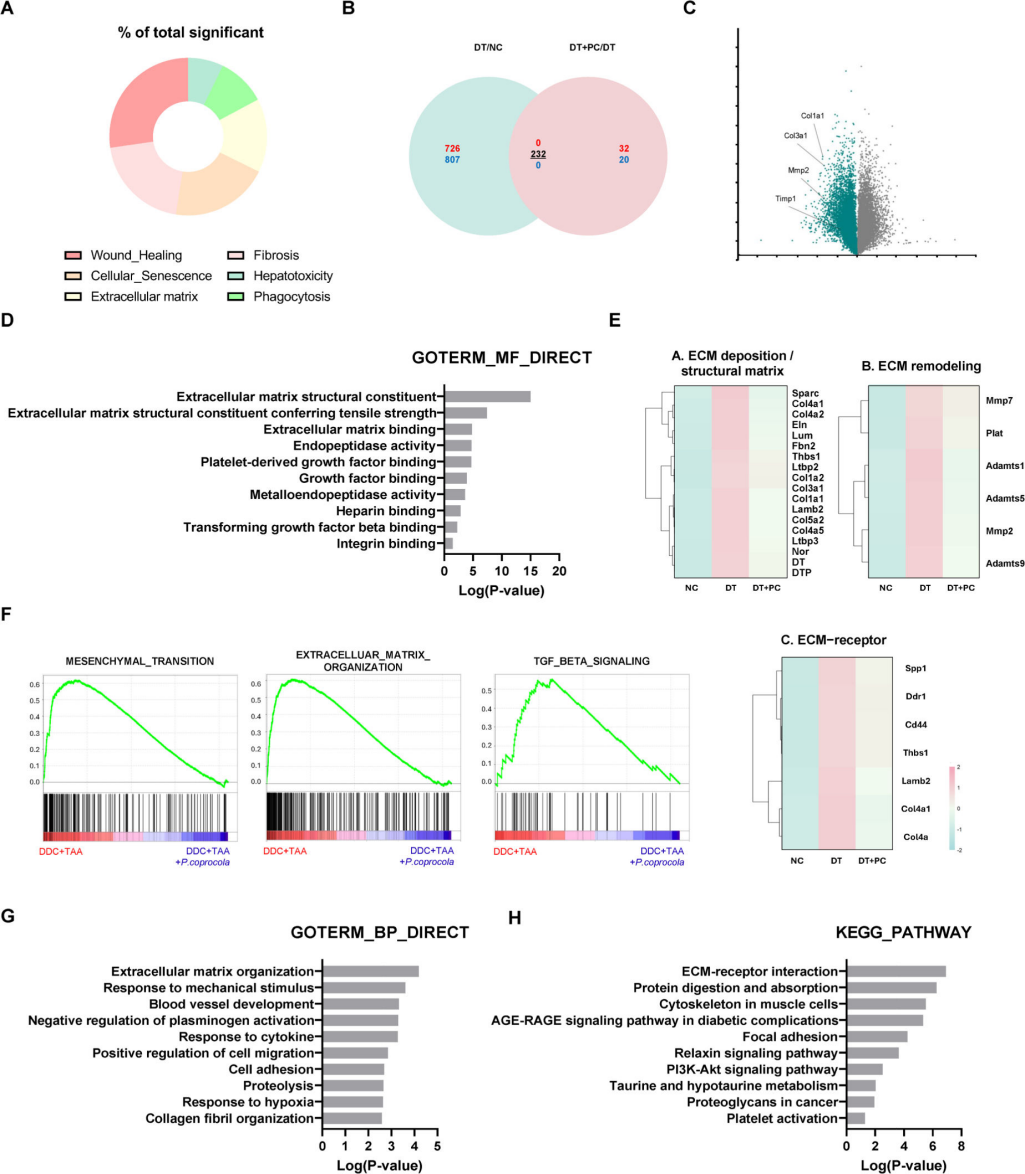
Transcriptomic analysis identifies extracellular matrix remodeling as a major altered pathway in advanced liver injury. (A) Functional categorization of significantly altered genes based on GO terms, showing the proportion of enriched biological processes. (B) Venn diagram illustrating the overlap of DEGs between DT vs NC and DT + PC vs DT comparisons. (C) Volcano plot showing DEGs between DT and DT + PC groups. (D) GOTERM_MF_DIRECT enrichment analysis of DEGs. (E) Heatmaps depicting expression patterns of genes involved in ECM deposition/structural matrix, ECM remodeling, and ECM–receptor interaction across NC, DT, and DT + PC groups. (F) GSEA plots for hallmark epithelial–mesenchymal transition, Reactome ECM organization, and hallmark TGF-*β* signaling pathways. (G) GOTERM_BP_DIRECT enrichment analysis of DEGs. (H) KEGG pathway enrichment analysis of DEGs. Abbreviations: NC, normal chow; DT, DDC + TAA; DT + PC, DDC + TAA plus *P. coprocola*; DEG, differentially expressed gene; GO, Gene Ontology; GSEA, gene set enrichment analysis; KEGG, Kyoto Encyclopedia of Genes and Genomes; ECM, extracellular matrix.

To identify potential mechanisms underlying the antifibrotic effects of *P. coprocola* in DDC + TAA–induced liver injury, we performed exploratory RNA sequencing of liver tissues from NC, DT, and DT + PC mice (Figure 6). Functional classification of differentially expressed genes revealed that pathways related to wound healing, fibrosis, cellular senescence, and ECM regulation comprised the largest proportion of significantly altered transcripts, indicating that ECM-centered processes represent the dominant transcriptional response to both injury and treatment (Figure 6A). To identify genes associated with disease-related transcriptional changes and their modulation by *P. coprocola*, we compared the DT/NC and DT + PC/DT contrasts using Venn diagram analysis. This analysis identified a subset of genes that were induced by DDC + TAA–mediated fibroinflammatory injury and suppressed following *P. coprocola* treatment (Figure 6B). This overlapping gene set was enriched for ECM–related transcripts, including *Col1a1*, *Col3a1*, *Mmp2*, and *Timp1*. Volcano plot analysis of the DT + PC/DT comparison further showed that these ECM-associated genes were significantly downregulated in DT + PC mice (Figure 6C).

To further characterize the functional identity of genes modulated by *P. coprocola*, we performed DAVID enrichment analysis on DT + PC/DT differentially expressed genes. Gene Ontology molecular function (GOTERM_MF) analysis revealed strong enrichment of terms associated with ECM structural constituents, ECM binding, growth factor binding, and integrin binding, indicating that *P. coprocola* broadly suppresses the molecular machinery required for ECM assembly and profibrotic signaling (Figure 6D). In parallel, Gene Ontology biological process (GOTERM_BP) analysis demonstrated significant enrichment of pathways involved in ECM organization, cell adhesion, cell migration, proteolysis, and collagen fibril assembly, reflecting a coordinated repression of tissue remodeling and fibrotic progression (Figure 5G). KEGG pathway analysis identified ECM–receptor interaction and focal adhesion as the dominant pathways affected by *P. coprocola* treatment (Figure 6H). Guided by these enrichment results, we extracted ECM-related genes from the overlapping DT-up/DTP-down gene set and examined their expression across NC, DT, and DT + PC groups. Heatmap analysis revealed that genes involved in ECM deposition and structural matrix formation, ECM remodeling, and ECM–receptor interactions were induced in DT livers and reduced in DT + PC livers (Figure 6E). Consistently, genes that were significantly upregulated in DT and downregulated in DT + PC were predominantly enriched within ECM organization and focal adhesion pathways, further supporting suppression of ECM-centered fibrotic programs by *P. coprocola* (Supplementary Fig. 4 A and B).

Consistent with these gene-level changes, gene set enrichment analysis (GSEA) showed that hallmark epithelial–mesenchymal transition (EMT), ECM organization, and TGF-*β* signaling pathways were enriched in DT livers and attenuated in DT + PC livers (Figure 6F). These transcriptomic analyzes indicate that *P. coprocola* is associated with suppression of ECM-related transcriptional programs in DDC + TAA–induced fibroinflammatory liver injury. Based on these findings, we next performed targeted validation of candidate pathways.

### Validation of extracellular matrix–focused antifibrotic effects of Phocaeicola coprocola in DDC + TAA–induced liver fibrosis

qPCR analysis of inflammatory and immune-associated markers, including *Tlr4*, *Tnf*, *Il6*, *Cd11c*, *Emr1, Ccr2* and *Ccl2* revealed only modest, non-significant reductions in DT + PC livers compared with DT controls (Supplementary Fig. 5). Consistent with this, expression of the core TGF-*β* signaling mediators *Smad2*, *Smad3*, and *Smad4* remained largely unchanged. Similarly, we observed a dissociation between pathway-level enrichment and gene-level validation for metabolic and stress-response programs. Although mRNA-seq and GSEA suggested modulation of bile acid and metabolic pathways (Figure 6F, H), targeted qPCR analysis revealed that hepatic expression of genes involved in bile acid synthesis and transport (*Nr1h4*, *Nr0b2*, *Cyp7a1*, *Abcb11*), oxidative stress response (*Nqo1*, *Gclc*), and endotoxin sensing (*Cd14*, *Lbp*) exhibited only modest, non-significant changes in DT + PC livers. Also, upstream fibrosis-associated genes such as *S100a4* and *Lox* showed limited transcriptional modulation(Supplementary Fig. 6). These findings indicate that *P. coprocola* does not broadly suppress hepatic inflammatory, metabolic, or upstream profibrotic signaling, but instead preferentially targets downstream fibrotic effector programs.

**Figure 7.**
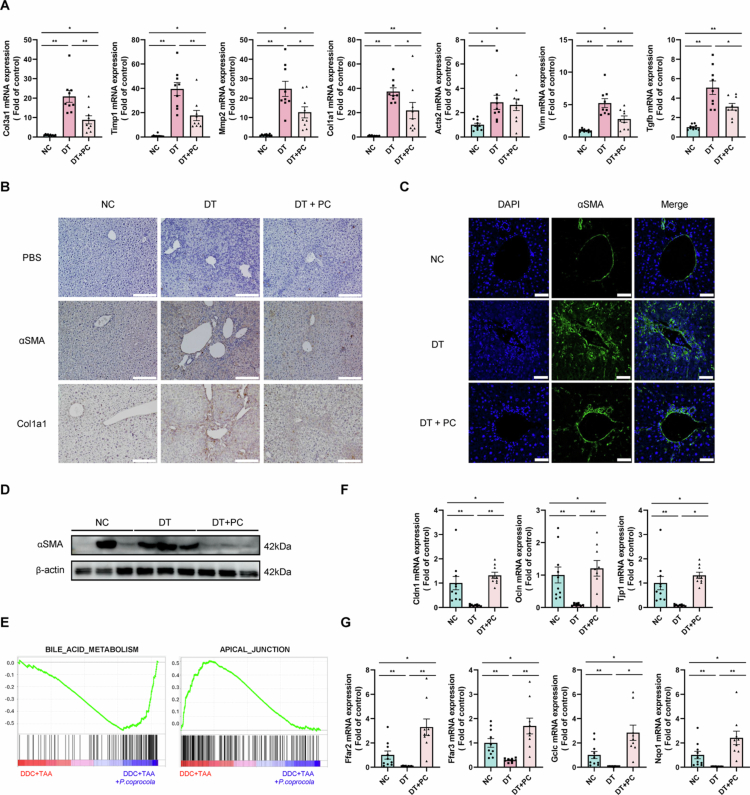
Validation of extracellular matrix–focused antifibrotic effects of *Phocaeicola coprocola* in DDC + TAA–induced liver fibrosis. (A) Hepatic mRNA expression of fibrosis- and ECM remodeling–related genes measured by qPCR (Col3a1, Timp1, Mmp2, Col1a1, Acta2, Vim, Tgfb) (B) IHC of liver sections for *α*-SMA and Col1a1. All images were acquired at 400 × magnification (scale bars, 50 μm). (C) IF of liver section for *α*-SMA (D) Western blot analysis of hepatic *α*-SMA protein expression. All images were acquired at 200 × magnification (scale bars, 50 μm). (E) GSEA plots for hallmark Bile acid metabolism and apical junction pathway. (F) Colonic mRNA expression of tight junction–related genes measured by qPCR (Cldn1, Ocln, Tjp1). (G) Colonic mRNA expression of SCFA receptor–and antioxidant response–related genes measured by qPCR (Ffar2, Ffar3, Gclc, Nqo1). Data are presented as mean ± SEM. Statistical significance was determined by ordinary one-way ANOVA followed by Tukey’s multiple comparisons test. *P < 0.05, **P < 0.01. Abbreviations: ECM, extracellular matrix; qPCR, quantitative polymerase chain reaction; GSEA, gene set enrichment analysis; *α*-SMA, alpha-smooth muscle actin, SCFA: short-chain fatty acid.

In contrast, downstream fibrotic effector programs were strongly modulated. qPCR analysis demonstrated that key extracellular matrix (ECM) genes, including *Col1a1*, *Col3a1*, *Acta2*, *Vim*, *Mmp2*, and *Timp1*, were significantly downregulated in DT + PC livers (Figure 7A). This transcriptional suppression was corroborated by histological and protein-level analyses. Immunohistochemical staining revealed markedly reduced periportal Col1a1 deposition and *α*-SMA expression in DT + PC mice compared with DT controls (Figure 7B). To further validate the specificity of *α*-SMA detection, immunofluorescence analysis confirmed selective localization of *α*-SMA to the portal tract and perivascular regions, with a marked reduction in DT + PC mice relative to DT controls (Figure 7C). Western blot analysis likewise demonstrated significantly reduced *α*-SMA protein expression following *P*. coprocola treatment (Figure 7D). Supporting these findings, GSEA demonstrated attenuation of extracellular matrix organization, epithelial–mesenchymal transition, and TGF-β–associated gene programs in DT + PC livers, whereas bile acid metabolism and epithelial junction pathways were comparatively less affected (Figure 7E).

Given the limited changes observed in hepatic upstream signaling pathways, we next examined whether *P. coprocola* might exert its antifibrotic effects through modulation of the gut–liver axis. qPCR analysis of colonic tissue revealed increased expression of epithelial barrier genes, including *Cldn1*, *Ocln*, and *Tjp1* (Zo-1), in DT + PC mice (Figure 7F). In addition, expression of the short-chain fatty acid receptors *Ffar2* and *Ffar3* and the antioxidant genes *Gclc* and *Nqo1* was also elevated (Figure 7G), indicating improved barrier integrity and enhanced microbial–host metabolic signaling. Collectively, these findings indicate that *P. coprocola* selectively attenuates fibrotic effector programs at the level of ECM remodeling, independent of broad suppression of hepatic inflammatory or upstream TGF-*β* signaling.

### Circulating extracellular matrix remodeling markers reflect the severity of human liver fibrosis

Given that ECM remodeling constitutes a final common pathway in fibrosis progression, we investigated whether this ECM-centered signature corresponds to clinically relevant fibrosis markers across different stages of chronic liver disease in human cohorts. Consistent with our murine findings where *Col3a1*, *Mmp2*, and *Timp1* were key responders to therapy, we found that their circulating human counterparts—PIIINP, MMP2, and TIMP1—reflected disease severity in patients with advanced fibrosis and cirrhosis.

**Figure 8.**
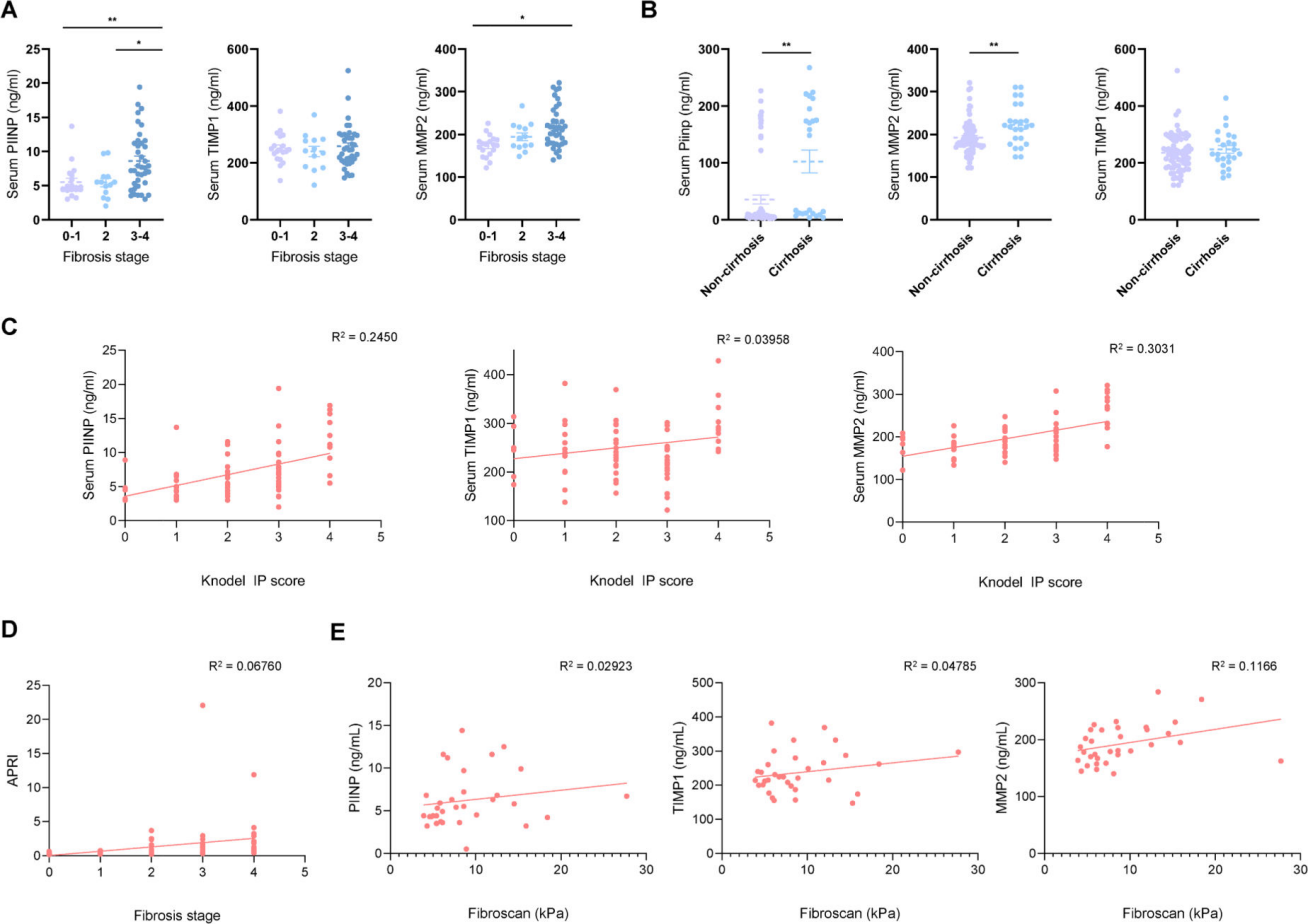
Circulating extracellular matrix remodeling markers reflect the severity of human liver fibrosis. (A) Serum levels of PIIINP, TIMP1, and MMP2 across fibrosis stages (F0–F4) in fibrosis patient samples. (B) Serum levels of PIIINP, TIMP1, and MMP2 comparing non-cirrhotic patients and cirrhotic patients. (C) Correlation analysis between serum PIIINP, TIMP1, and MMP2 levels and Knodell inflammatory scores in patient samples. (D) Correlation between the AST-to-platelet ratio (APR) and fibrosis stage in patient samples. (E) Correlation between serum MMP2 and TIMP1 levels and liver stiffness measured by FibroScan. (F) Correlation between serum PIIINP, TIMP1, and MMP2 levels and FibroScan scores. Data are presented as individual values with regression lines where applicable. Statistical significance across fibrosis stages was determined using the Kruskal–Wallis test followed by Dunn’s multiple comparisons test. Correlation analyses were performed using nonlinear or linear regression, as appropriate. Abbreviations: PIIINP, procollagen type III N-terminal peptide; MMP2, matrix metalloproteinase 2; TIMP1, tissue inhibitor of metalloproteinases 1; APRI, aminotransferase–to–platelet ratio index; ECM, extracellular matrix.

Specifically, serum PIIINP and MMP2 levels showed a progressive increase across fibrosis stages (F0–F4) (Figure 8 A) and were significantly higher in patients with cirrhosis compared with those without cirrhosis (Figure 8B). While serum TIMP1 levels showed less distinct stratification across fibrosis stages, they remained positively associated with histological disease activity. In parallel, the AST-to-platelet ratio (APR), a widely used noninvasive surrogate marker of liver fibrosis, demonstrated a progressive increase with advancing fibrosis stage (Figure 8D). Although inter-individual variability was observed, particularly at higher stages, APR values tended to be elevated in patients with more advanced fibrosis, supporting its relevance as a clinical indicator of fibrosis burden.

Consistent with this, serum TIMP1 levels were positively correlated with Knodell inflammatory scores (Figure 8C), indicating that circulating TIMP1 reflects active tissue remodeling and inflammatory injury in human patients. Furthermore, all three ECM-related markers (PIIINP, MMP2, and TIMP1) showed positive trends with liver stiffness as measured by FibroScan (Figure 8E).

In conclusion, the elevation of these markers in human disease and their correlation with clinical severity validate the translational importance of the ECM targets identified in mice. The ability of *P. coprocola* to selectively suppress these specific fibrogenic effectors underscores its potential as a precision microbiome therapeutic for advanced liver fibrosis. This supports emerging concepts that microbial effects on host physiology can occur independently of stable colonization, expanding the current understanding of microbiome-based therapeutics.

## Discussion

Chronic liver diseases, including metabolic dysfunction–associated steatotic liver disease (MASLD), frequently progress to liver fibrosis, for which effective antifibrotic therapies remain limited.^1^ ^,^ ^7^ In this study, we identify *Phocaeicola coprocola* as a selective modulator of the gut–liver axis that exhibits antifibrotic activity across two mechanistically distinct murine models of liver disease. Notably, rather than broadly suppressing hepatic inflammation or upstream signaling pathways, *P. coprocola* primarily targets extracellular matrix (ECM) production and remodeling, a key terminal process in fibrogenesis.^4^ ^,^ ^22^

In the WD-induced MASLD model*, P. coprocola* supplementation attenuated hepatic steatosis, reduced lipogenic gene expression (*Fasn, Scd1, Acaca*),^23‐25^ and improved metabolic and histological parameters. In contrast, in the DDC + TAA model of advanced fibrosis, cholestatic markers (ALP and GGT) were significantly reduced despite modest changes in aminotransferases, consistent with the cholestatic nature of this model. While canonical inflammatory mediators and upstream TGF-β–Smad signaling remained largely unchanged,^26‐30^ fibrogenic genes (*Col1a1, Col3a1, Acta2 and Vim*) and ECM remodeling genes (*Mmp2 and Timp1*) were selectively suppressed,^31‐33^ indicating that *P. coprocola* primarily acts at the level of ECM-associated fibrogenic effector programs rather than upstream inflammatory pathways. These findings extend previous studies on the gut–liver axis, which have largely emphasized inflammatory and metabolic pathways, by highlighting ECM remodeling as a distinct and potentially targetable downstream effector mechanism in liver fibrosis. It should be noted that the histological pattern of fibrosis differs between the two models employed in this study. WD-induced fibrosis is typically pericellular/perisinusoidal, driven by lipotoxicity-mediated HSC activation, whereas the DDC + TAA combination model produces a more complex fibroinflammatory phenotype: DDC pretreatment induces cholangitis and periductal fibrosis through bile duct injury, while TAA drives hepatocellular injury and bridging fibrosis, collectively recapitulating features of advanced human cirrhosis. The consistent efficacy of *P. coprocola* across both models supports the generalizability of its therapeutic effects across distinct fibrosis etiologies. Importantly, *P. coprocola* supplementation in healthy mice did not induce hepatic or metabolic abnormalities. Although a modest increase in ALT was observed, this occurred in the absence of histopathological changes and with normal AST, ALP, GGT, and albumin levels, supporting its safety under physiological conditions. Additionally, colonic expression of epithelial barrier–related genes (*Cldn1*)^34^ and short-chain fatty acid receptors (*Ffar2* and *Ffar3*)^35^ ^,^ ^36^ was significantly increased, indicating enhanced intestinal barrier function and host–microbial metabolic responsiveness. The antifibrotic effects of *P. coprocola* were observed even in the absence of detectable gut colonization in the DDC + TAA model, suggesting that stable engraftment is not required for its activity. To further investigate the molecular mechanisms underlying these antifibrotic effects, transcriptomic analysis was performed in the DDC + TAA model, which exhibits a more advanced fibrotic phenotype (F3–F4) with robust ECM remodeling compared to the WD-induced MASLD model. This model provides a greater dynamic range for detecting fibrosis-associated transcriptional programs, enabling more effective identification of ECM-related pathways modulated by *P. coprocola*. This is consistent with emerging evidence that microbial-derived signals can modulate host physiology independently of persistent colonization.^37‐39^ Given that these effects converge on ECM remodeling, we next assessed the clinical relevance of this mechanism in human liver disease. Circulating markers of matrix remodeling, including PIIINP, MMP2, and TIMP1,^40^ ^,^ ^41^ were associated with fibrosis severity. The concordance between circulating ECM remodeling markers and the ECM-related genes suppressed in *P. coprocola*–treated mice supports the clinical relevance of ECM-targeted pathways in human liver fibrosis.^42^

This study has several limitations. First, although *P. coprocola* demonstrated clear antifibrotic efficacy, it did not achieve stable colonization in the DDC + TAA model, suggesting that its therapeutic effects may be mediated through transient microbial activity or metabolite-driven mechanisms rather than persistent microbiome restructuring. Direct mechanistic validation using fecal microbiota transplantation (FMT), together with targeted identification of *P. coprocola*-derived bioactive metabolites, will be essential to define the precise mechanisms underlying these effects and represents an important direction for future investigation.^43^ ^,^ ^44^ In addition, the clinical cohorts were not fully age-matched, and age may represent a potential confounding factor, as it is independently associated with both liver disease progression and gut microbiota composition. Finally, as this study primarily reflects a prevention model, future studies should evaluate therapeutic efficacy in established fibrosis and assess durability of response. Furthermore, as *P. coprocola* is not currently recognized under established probiotic regulatory frameworks (FDA GRAS or EFSA QPS), comprehensive safety evaluation will be required for clinical translation.^45‐48^

In conclusion, *P. coprocola* selectively targets ECM remodeling during liver fibrosis, uncoupling fibrotic output from upstream inflammatory signaling. These findings identify ECM remodeling as a key downstream effector of the gut–liver axis and provide a rationale for exploring *P. coprocola* as a microbiome-based therapeutic approach for liver fibrosis across multiple disease etiologies.

## Materials and methods

### Patient

A prospective cohort study was conducted between July 2018 and August 2021. Written informed consent was obtained from all participants prior to enrollment. The study was registered in the public clinical trial registry at ClinicalTrials.gov (Identifier: NCT04339725). A total of 151 individuals were enrolled and classified into three groups: 54 healthy controls, 78 patients diagnosed with metabolic dysfunction-associated steatotic liver disease (MASLD), and 19 patients with advanced liver disease, including liver cirrhosis. Detailed clinical and biochemical characteristics of the study population are provided in Supplementary Table 2.

MASLD was defined according to the consensus criteria for metabolic dysfunction–associated steatotic liver disease,^49^ ^,^ ^50^ requiring the presence of hepatic steatosis in the context of cardiometabolic risk factors. In our cohort, diagnosis was based on clinical and biochemical evidence of metabolic dysfunction and liver injury, supported by elevated or abnormal serum transaminases (AST and ALT). Where available, histological assessment from liver biopsy was used to evaluate steatosis, inflammation, and fibrosis according to the NAFLD Activity Score (NAS) and fibrosis staging system.

Healthy controls were defined as individuals without cardiometabolic risk factors, with normal liver biochemistry (AST, ALT, GGT within reference ranges), and no history of liver disease or metabolic comorbidities. Although imaging studies were not systematically performed, the absence of biochemical and metabolic abnormalities makes undiagnosed liver disease unlikely in this cohort.

Exclusion criteria included a history of alcoholic liver disease, drug-induced liver injury, autoimmune liver disease, and pregnancy. All participants were aged ≥ 20 years and had a body mass index (BMI) > 23 kg/m^2^. Healthy controls were recruited by individuals undergoing routine health check-ups. All participants underwent blood chemistry testing, including measurement of aspartate aminotransferase (AST), alanine aminotransferase (ALT), creatinine, total cholesterol, *γ*-glutamyl transferase (γGTP), triglycerides, and high-density lipoprotein (HDL) cholesterol levels. Fecal samples were collected from all participants and immediately stored at –80 °C until further analysis.

In addition, an independent cohort of patients with chronic liver disease who underwent liver biopsy for fibrosis staging was used for serum biomarker analysis (Figure 7). These patients were stratified according to histological fibrosis stage (Stage 0–4), and patients with hepatocellular carcinoma (HCC) were excluded. Detailed characteristics of this cohort are provided in Supplementary Table 3. Baseline characteristics are presented as mean ± standard deviation (SD) and were compared between groups using one-way ANOVA for continuous variables. *P*-values < 0.05 were considered statistically significant.

### Microbiome analysis based on 16S rRNA gene sequencing

Genomic DNA was extracted from fecal samples using the QIAamp DNA Stool Mini Kit (QIAGEN, Germany) following the manufacturer’s protocol. The V3–V4 hypervariable regions of the bacterial 16S rRNA gene were amplified using barcoded universal primers. PCR amplification was performed under the following cycling conditions: initial denaturation at 95 °C for 5 min; 20 cycles of 95 °C for 30 s, 55 °C for 30 s, and 72 °C for 30 s; and a final extension at 72 °C for 10 min. Amplicons were purified using the Agencourt AMPure XP system (Beckman Colter) and quantified with PicoGreen and qPCR.

Pooled libraries were sequenced on the Illumina MiSeq platform using paired-end 150 bp reads. Sequence data were analyzed using the EzBioCloud 16S-based Microbial Taxonomic Profiling platform (CJ Bioscience, Republic of Korea). Analyses included taxonomic assignment using the EzBioCloud reference database, calculation of alpha- and beta-diversity indices, and differential abundance testing including LEfSe.

### Animal experiment

All animal experiments were performed in accordance with institutional and national guidelines for the care and use of laboratory animals and were approved by the Animal Experiment Management Committee of Hallym University College of Medicine (IACUC No. 2022-60). Five-week-old male specific pathogen-free C57BL/6J mice were obtained from Dooyeol Biotech (Seoul, Republic of Korea).

Animals were maintained individually in microisolator cages under controlled environmental conditions (22 ± 2 °C, 12-h light/dark cycle) with unrestricted access to food and water. Mice were allowed to acclimate for one week on a normal chow diet prior to the start of experimental procedures.

To induce metabolic dysfunction–associated steatotic liver disease (MASLD), mice were fed a Western diet (WD; TD.88137, Envigo/Inotiv; 42% kcal from fat [21.2% by weight], primarily derived from anhydrous milkfat rich in saturated fatty acids [palmitic acid C16:0, myristic acid C14:0, and stearic acid C18:0] and monounsaturated fatty acids [oleic acid C18:1]; 42.7% kcal from carbohydrate [48.5% by weight]; 15.2% kcal from protein [17.3% by weight]; 0.2% cholesterol by weight) for 18 weeks. After 9 weeks of WD feeding, animals were randomly assigned to treatment groups and administered either vehicle or *Phocaeicola coprocola* (1 × 10^9^ CFU suspended in PBS) via oral gavage three times per week for the remaining 9 weeks while continuing the WD regimen (NC, *n* = 10; WD, *n* = 10; WD + PC, *n* = 9).

For induction of advanced fibroinflammatory liver injury, mice were provided standard rodent chow (Teklad Global 18% Protein Rodent Diet, 2018; Envigo/Inotiv; protein 18.6% by weight; fat 6.2% by weight, primarily derived from soybean oil rich in polyunsaturated fatty acids [linoleic acid C18:2 and alpha-linolenic acid C18:3] and monounsaturated fatty acids [oleic acid C18:1]) supplemented with 0.05% w/w 3,5-diethoxycarbonyl-1,4-dihydrocollidine (DDC), custom-prepared by DooYeol Biotech (Seoul, Republic of Korea), in combination with thioacetamide (TAA; 200 mg/L) administered via drinking water for 10 weeks. During this period, animals received either vehicle or *P. coprocola* (1 × 10^9^ CFU in PBS) by oral gavage three times weekly (NC, *n* = 10; DT, *n* = 9; DT + PC, *n* = 9).

To assess the effects of *P. coprocola* under physiological conditions, an additional group of mice maintained on normal chow diet received either vehicle or *P. coprocola* (1 × 10^9^ CFU in PBS) via oral gavage daily for 3 weeks (NC, *n* = 3; NC + PC, *n* = 3).

The dose of *P. coprocola* used in all animal experiments (1 × 10^9^ CFU/mouse/day) was determined based on interspecies dose conversion from human clinical reference data using the body surface area normalization method.^51^ Briefly, a human reference dose of 1.0 × 10^11^ CFU/day (body weight 60 kg; Km = 37) was converted to a mouse-equivalent dose as follows: mouse dose = human dose × (Km mouse/Km human) × (mouse body weight/human body weight) = 1.0 × 10^11^ × (3/37) × (0.02/60) = 4.11 × 10^8^ CFU/day. A slightly higher dose of 1.0 × 10^9^ CFU/day was selected to account for potential loss of probiotic viability during gavage preparation and administration.

At the end of the experimental period, mice were sacrificed under approved protocols, and liver and colon tissues were collected for subsequent histological, molecular, and biochemical analyses.^52^

### Liver histological analysis

Liver tissues were harvested, fixed in 10% neutral-buffered formalin, and embedded in paraffin. Tissue sections were stained with hematoxylin and eosin (H&E), Sirius Red, and Masson’s trichrome to assess hepatic injury, fibrosis, and overall liver architecture. Histological scoring was performed using the NAFLD Activity Score (NAS) system developed by the Nonalcoholic Steatohepatitis Clinical Research Network (NASH CRN), which integrates three components: steatosis (0–3), lobular inflammation (0–3), and hepatocellular ballooning (0–2). The scoring criteria were as follows: Steatosis: (0: <5% of hepatocytes affected; 1: 5–33%; 2: 34–66%; 3: >66%). Lobular inflammation: (0: no foci; 1: <2 inflammatory foci per 200 × field; 2: 2–4 foci per 200 × field; 3: >4 foci per 200 × field). Hepatocellular ballooning: (0: none; 1: few ballooned cells; 2: many ballooned cells and/or prominent ballooning). Fibrosis was assessed independently using the NASH CRN fibrosis staging system: Fibrosis staging: (0: no fibrosis; 1: zone 3 perisinusoidal fibrosis only; 2: zone 3 and portal/periportal fibrosis; 3: bridging fibrosis; 4: cirrhosis). All histopathological assessments were performed by a board-certified liver pathologist blinded to group allocation. Quantitative fibrosis areas from Sirius Red and Masson’s trichrome staining were also analyzed using ImageJ software for morphometric evaluation of collagen deposition.

### Blood chemistry test

Animal serum samples were allowed to coagulate overnight at 4 °C, then centrifugation at 2000g for 20 min room temperature. The resulting supernatant was collected, aliquoted, and stored at −80 °C until analysis. Serum biochemical markers, including alanine aminotransferase (ALT), alkaline phosphatase (ALP), gamma-glutamyl transferase (GGT), total cholesterol, low-density lipoprotein (LDL), bilirubin (BIL), and albumin (Alb), were measured using an automated clinical chemistry analyzer (KoneLab 20; Thermo Fisher Scientific).

### Immunohistochemistry (IHC) staining

Paraffin-embedded liver tissue sections (4 μm thick) were deparaffinized in xylene, rehydrated through a graded ethanol series, and subjected to heat-induced antigen retrieval in citrate buffer (pH 6.0) at 95 °C for 10 minutes. Endogenous peroxidase activity was blocked by incubation in 3% hydrogen peroxide for 5 minutes at room temperature. Tissue sections were then blocked with 5% bovine serum albumin (BSA) in PBS for 60 minutes at room temperature to reduce non-specific binding. Slides were incubated overnight at 4 °C with the following primary antibodies: anti-collagen type I alpha 1 chain (Col1a1, ab21286, Abcam, UK; 1:200 dilution) and anti-alpha smooth muscle actin (*α*-SMA, ab124964, Abcam, UK; 1:100 dilution). The LSAB + System-HRP kit (Dako, USA) was used for detection according to the manufacturer’s protocol. Briefly, sections were incubated with a biotinylated secondary antibody followed by streptavidin-conjugated horseradish peroxidase (HRP). Signal development was performed using 3,3′-diaminobenzidine (DAB) as the chromogen, and nuclei were counterstained with hematoxylin. After dehydration and mounting, images were acquired using a light microscope. Quantitative analysis of positively stained areas was conducted using ImageJ software (NIH, USA).

### Immunofluorescence staining

Paraffin-embedded liver tissues were prepared after fixation in 4% paraformaldehyde (Biosesang, Seoul, Korea) and sectioned at a thickness of 10 μm. Tissue sections were deparaffinized, rehydrated through a graded ethanol series, and subjected to heat-induced antigen retrieval in 10 mM citrate buffer (pH 6.0) using a pressure cooker for 10 min.

After antigen retrieval, sections were incubated for 1 h at room temperature in PBS containing 5% bovine serum albumin (BSA) and 0.3% Triton X-100 to block nonspecific binding. The sections were then incubated overnight at 4 °C with rabbit anti-*α*-SMA antibody (ab124964, Abcam, UK; 1:100 dilution). diluted in the same blocking solution. Following PBS washes, sections were incubated with Alexa Fluor 633 goat anti-rabbit IgG (H + L) secondary antibody( A-21070, Invitrogen, USA;1:400) for 1 h at room temperature in the dark. Finally, the slides were mounted using Fluoro-Gel II with DAPI (Electron Microscopy Sciences, USA).

Fluorescence images were obtained using an LSM 900 confocal microscope equipped with Airyscan 2 (Carl Zeiss, Jena, Germany). Image acquisition and processing were performed using ZEN Blue software (Carl Zeiss), and brightness and contrast were adjusted uniformly across all samples.

### Quantitative reverse transcription polymerase chain reaction (qRT-PCR)

Total RNA was isolated from tissue samples using TRIzol reagent (Thermo Fisher Scientific, Waltham, MA, USA) following the recommended protocol. RNA quantity and purity were evaluated spectrophotometrically, and 2 μg of total RNA was subsequently reverse-transcribed into complementary DNA (cDNA) using the High-Capacity cDNA Reverse Transcription Kit (Thermo Fisher Scientific). Quantitative real-time PCR was conducted using SYBR Green Master Mix (Thermo Fisher Scientific) on a LightCycler® 480 Real-Time PCR System (Roche Diagnostics)^53^. Primer sequences are provided in Supplementary Table S1.

### mRNA sequencing

mRNA was extracted from representative mouse liver tissues (*n* = 2 biological replicates per group, exploratory analysis) selected for transcriptomic profiling. RNA integrity and concentration were assessed using a Qubit fluorometer (Thermo Fisher Scientific, Waltham, MA, USA) and an Agilent 2100 Bioanalyzer with the RNA 6000 Nano Kit (Agilent Technologies, Santa Clara, CA, USA). Sequencing libraries were constructed using the QuantSeq 3′ mRNA-Seq Library Prep Kit (Lexogen, Vienna, Austria) in accordance with the manufacturer's protocol. High-throughput sequencing was performed on the Illumina NextSeq 500 platform to generate single-end 75 bp reads.

### Bioinformatic analysis

Primary data processing was conducted by a commercial service provider (eBiogen Inc., Seoul, Republic of Korea) using the in-house ExDEGA platform. As raw FASTQ files were not retained, downstream statistical analyses were performed using gene-level count matrices derived from representative samples. Briefly, sequencing reads were aligned to the mouse reference genome (GRCm39) using Bowtie2 (Langmead and Salzberg, 2012), and gene-level read counts were quantified using Bedtools (Quinlan, 2010).

For differential expression analysis, raw counts were normalized using the Trimmed Mean of M-values (TMM) method and converted to counts per million (CPM) followed by log2 transformation. Statistical significance of differential expression was determined using the edgeR package in R (Bioconductor). Functional enrichment and pathway trend analyses were performed using the DAVID database (https://david.ncifcrf.gov), focusing on Gene Ontology (GO) terms and Kyoto Encyclopedia of Genes and Genomes (KEGG) pathways.

Furthermore, Gene Set Enrichment Analysis (GSEA) was performed to assess the enrichment of specific biological signatures, including hallmark gene sets and canonical pathways.

### Strain preparation

*Phocaeicola coprocola* was identified based on full-length 16S rRNA gene sequencing. The isolate was cultured for 48 h at 37 °C under strictly anaerobic conditions on Tryptic Soy Agar (TSA; Kisan Bio, Korea) supplemented with defibrinated sheep blood (Kisan Bio, Korea).

For animal administration, bacterial cells were harvested, washed, and resuspended in sterile 1 × phosphate-buffered saline (PBS). The bacterial suspension was adjusted to a final concentration of 1 × 10^9^ CFU/mL and was orally administered to mice three times per week.

### Culturomics and phenotypic profiling of fecal isolates

Fecal samples from mice in each dietary group (Normal diet, Western diet, Western diet + *P. coprocola*) were pooled and subjected to culturomics-based bacterial isolation. Briefly, fecal material was suspended in sterile saline (1 g/mL), serially diluted, and plated on modified Gifu Anaerobic Medium (mGAM) agar without additional supplements. After anaerobic incubation, colonies were selected based on distinct morphological characteristics. Bacterial isolates were then identified using matrix-assisted laser desorption ionization time-of-flight mass spectrometry (MALDI-TOF MS) on a Biotyper Sirius system (Bruker Daltonics, Bremen, Germany).

To assess strain-level phenotypic variation, ten Enterococcus faecalis isolates from each group were selected and subjected to Fourier-transform infrared (FT-IR) spectroscopy. Spectral data were acquired using an FT-IR analyzer and principal component analysis (PCA) was performed to visualize clustering based on phenotypic profiles.

### Western blot

Liver tissues were homogenized in RIPA lysis buffer (Thermo Scientific) and incubated on ice for 1 h. Lysates were centrifuged at 14,000 rpm for 10 min at 4 °C, and the supernatants were collected. Protein concentrations were determined using a bicinchoninic acid (BCA) protein assay kit (Thermo Scientific), and samples were stored at −20 °C until use.

Equal amounts of protein were mixed with protein sample buffer (Elpis Biotech), separated by SDS–polyacrylamide gel electrophoresis (SDS–PAGE), and transferred onto polyvinylidene difluoride (PVDF) membranes. Following transfer, membranes were incubated for 1 hour at room temperature in TBST containing either 5% bovine serum albumin (BSA) or 5% skim milk to minimize nonspecific antibody binding.

Membranes were incubated overnight at 4 °C with primary antibodies against *α*-smooth muscle actin (*α*-SMA; ab124964; Abcam) and beta-actin (*β*-actin; 3700S; Cell Signaling Technology) as a loading control. After washing three times with TBST (5 min each), membranes were incubated with secondary antibodies for 1 h at room temperature. Following three additional washes with TBST, protein bands were visualized using Immobilon Forte Western HRP substrate (MERCK) and detected using an Amersham Imager 680 system (Amersham).

### Laboratory tests

Serologic markers associated with liver fibrosis were measured, including indicators related to extracellular matrix turnover. All serum samples were analyzed in a single laboratory under the same conditions. Serum levels of MMP2 and TIMP1 were quantified using Quantikine® immunoassay kits (R&D Systems, Minneapolis, MN, USA), with reference ranges of 0.78–50 ng/mL and 0.05–100 ng/mL, respectively. The concentration of procollagen type III *N*-terminal peptide (PIIINP) was determined using the UniQ PIIINP radioimmunoassay kit (Orion Diagnostica, Espoo, Finland), with a reference range of 2.3–6.4 μg/L. The AST-to-platelet ratio index (APRI) was calculated using the previously established formula: (AST/upper limit of normal)/platelet count (10^9^/L) × 100.^54^

### Histological examination and analysis

Liver tissue samples were obtained through percutaneous biopsy using an 18-gauge needle. The collected specimens were immediately fixed in 10% neutral-buffered formalin, processed using standard paraffin-embedding procedures, and sectioned for histological evaluation. Tissue sections were stained with hematoxylin and eosin for general morphology, Masson’s trichrome for collagen deposition, and reticulin staining to assess architectural changes.

All biopsy materials were evaluated at a single pathology center by two experienced hepatopathologists who were blinded to the clinical information. The degree of fibrosis was graded according to the METAVIR scoring system, ranging from F0 (no fibrosis) to F4 (established cirrhosis), based on the extent of fibrotic expansion and septa formation. In cases where the initial interpretations differed, the final stage was determined by joint review and consensus using a multi-headed microscope.^54^

### Statistical analysis

All statistical analyses were carried out using GraphPad Prism software (version 9.0.0; GraphPad Software, San Diego, CA, USA). Data are presented as the mean ± standard error of the mean (SEM). For human cohort data, differences between two groups were evaluated using the Mann–Whitney U test. Comparisons among multiple experimental groups were analyzed using ordinary one-way analysis of variance (ANOVA) followed by Tukey’s post hoc test.^52^ Differences across fibrosis stages were assessed using the Kruskal–Wallis test with Dunn’s multiple comparisons test. Correlations between variables were examined using linear or nonlinear regression models, as appropriate. A *p*-value of less than 0.05 was considered statistically significant. Significance levels are indicated as *P < 0.05, **P < 0.01, and ***P < 0.001.

## Supplementary Material

Supplementary.docx

## Data Availability

The raw sequencing data (16S rRNA gene) generated in this study have been deposited in the NCBI Sequence Read Archive (SRA) under BioProject accession number PRJNA1417901. The processed taxonomic abundance tables used for statistical analyses and figure generation are publicly available in the Zenodo repository at https://doi.org/10.5281/zenodo.18464089. The authors confirm that the data supporting the findings of this study are available within the article and its supplementary materials.
