# Supplementary material for: Phocaeicola coprocola attenuates liver fibrosis by modulating extracellular matrix remodeling
Source: Gut Microbes. 2026 Aug 17;18(1):2718623. doi: 10.1080/19490976.2026.2718623 (PMC13483468; doi:10.1080/19490976.2026.2718623)
Supplement: Supplementary file 1 [file KGMI_A_2718623_SM9877.docx]

***Phocaeicola coprocola* attenuates liver fibrosis by modulating extracellular matrix remodeling**

Kyeong Jin Lee^a†^, Jeong Ha Park^a†^, Hyunjoon Park^a^, Min Ju Kim^a^, Ho yeong Yoon^a^, Jeong Min Kim^a^, Sang Hak Han^b^, Ki Tae Suk^a*^

*^a^ Institute for Liver and Digestive Diseases, Hallym University, Chuncheon 24253, Republic of Korea;^b^ Department of Pathology, Hallym University College of Medicine, Chuncheon 24253, Republic of Korea*

^†*^These authors equally contribute

Ki Tae Suk, M.D., Ph.D.

Department of Internal Medicine, Chuncheon Sacred Heart Hospital Hallym University

Gyo-dong, Chuncheon, South Korea. Zip 24253

Telephone No.: +82-33240-5826, Fax No.: +82-33-241-8064

E-mail address: [ktsuk@hallym.ac.kr](mailto:ktsuk@hallym.ac.kr)

**Supplementary Figure 1. Gut microbiota diversity and taxonomic composition in a Western diet–induced MASLD model.** (A) Alpha diversity indices. (B) Relative abundance of Firmicutes and Bacteroidetes.(C) Relative abundance of gut microbiota at the family level

**Supplementary Figure 2. *P. coprocola* partially reshapes gut microbial composition under Western diet conditions** (A) Culturomics workflow and bacterial strain isolation from pooled fecal samples of each group. (B) Venn diagram showing shared and unique bacterial strains among groups. (C) Isolation frequencies of Enterococcus species across experimental groups. Bars represent the quantitative abundance of individual Enterococcus species determined by culture-based isolation. (D) Relative abundance of Enterococcus faecalis and Enterococcus faecium isolates (E) Strain-level phenotypic clustering of E. faecalis isolates via FT-IR and PCA**.**

**Supplementary Figure 3. Gut microbiota diversity and taxonomic composition in the DDC+TAA–induced liver fibrosis model.** (A) Alpha diversity indices. (B) Relative abundance of Firmicutes and Bacteroidetes. (C) Relative abundance of gut microbiota at the family level

**Supplementary Figure 4. Expression patterns of extracellular matrix–related genes reversed by *P. coprocola*.** (A) Heatmap showing representative genes involved in reactome extracellular matrix (ECM) organization that were significantly upregulated in the DT group compared with NC and subsequently downregulated in the DT+PC group compared with DT (DT up, DT+PC down). (B) Heatmap showing representative genes associated with the KEGG focal adhesion pathway exhibiting the same expression pattern (DT up, DT+PC down). Genes were selected based on pathway enrichment analyses using DAVID and KEGG. Heatmaps display normalized gene expression values representing group means for NC, DT, and DT+PC samples.

**Supplementary Figure 5. Limited effects of PC treatment on inflammatory macrophage markers and canonical SMAD signaling.** (A–B) Hepatic mRNA expression of inflammatory and macrophage-associated markers measured by qPCR (*Tlr4, Tnf, Il6, Emr1, Cd11b, Ccl2,* and *Ccr2*). (C) Hepatic mRNA expression of genes involved in TGF-β signaling measured by qPCR (*Smad2, Smad3,* and *Smad4*).

**Supplementary Figure 6. Hepatic gene expression analysis related to bile acid metabolism, oxidative stress, and endotoxin signaling.** (A–B) Hepatic mRNA expression of genes involved in bile acid metabolism and oxidative stress measured by qPCR (*Nr1h4, Nr0b2, Cyp7a1, Abcb11, Nqo1,* and *Gclc*). (C) Hepatic mRNA expression of endotoxin sensing and upstream fibrosis-associated genes measured by qPCR (*Cd14, Lbp, S100a4,* and *Lox*).

**Supplementary Table S1.** Primer sequences for qRT-PCR analysis

| Gene | Forward primer (5’-3’) | | Reverse primer (5’-3’) |
| --- | --- | --- | --- |
| *18s* | | GTAACCCGTTGAACCCCATT | CCATCCAATCGGTAGTAGCG |
| *Ocln* | | ACCCGAAGAAAGATGGATCG | CATAGTCAGATGGGGGTGGA |
| *Cldn*  *Tjp1* | | CGGGCAGATACAGTGCAAAG  TGGGAACAGCACACAGTGAC | ACTTCATGCCAATGGTGGAC  GCTGGCCCTTTTAACAC |
| *Tnf*  *Ccl5*  *Ccl2*  *Scd1* | | CCCCAAAGGGATGAGAAGTT  CCAGCAGTCGTCTTTGTCAC  TCTGGACCCATTCCTTCTTG  GGCAGTTCTGAGGTGATTAGAG | CACTTGGTGGTTTGCTACGA  CTCTGGGTTGGCACACACTT  AGGTCCCTGTCATGCTTCTG  GTCTCTGGGAAGAGCAATGTAG |
| *Col1a1*  *Col3al*  *Tgfβ*  *Timp1*  *Mmp2*  *Mmp8*  *Vim*  *Tlr4* | | CATGTTCAGCTTTGTGGACCT  ACTTCCAACCCAGGTCCTTC  CAGCAAAGGCCTTCGTAA  GTCCCAGACATCAGGGAGTAA  GTTCAACGGTCGGGAATACA  TCAACCAGGCCAAGGTATTG  CGGAAAGTGGAATCCTTGCAGG  GCTTACACCACCTCTCAAACT | GCAGCTGACTTCAGGGATGT  GTGGAAATCAACGGGATCAG  TGGCTGAACAGGGAAACACT  TCGGATACTTCAGCGTCAGGA  GCCATACTTGCCATCCTTCT  ATGAGCAGCCACGAGAAATAG  AGCAGTGAGGTCAGGCTTGGAA  ACAGCCACCAGATTCTCTAAAC |
| *Pparα* | | AGAAGTTGCAGGAGGGGATT | TCGGACTCGGTCTTCTTGAT |
| *Cpt1α*  *Acox1* | | GATGTGGACCTGCATTCCTT  CACGGCTATTCTCACAGCAG | TCTTGTAATGTGCGAGCTG  CAGGCTGTTAATGTCCACCA |
| *Srebp1* | | TACTTCTTGTGGCCCGTACC | TCAGGTCATGTTGGAAACCA |
| *Cd36* | | GGAGTGCTGGATTAGTGGTTAG | GCTGTGAGCAGACGTATAGAAG |
| *Pparg* | | CCAAATAGCTTTATCTGGTGTTTC | CGTTGCTACATTGTCTCGC |
| *Fas* | | AAGCGGTCTGGAAAGCTGAA | AGGCTGGGTTGATACCTCCA |
| *Acaca*  *Mlxipl*  *Fabp5*  *Acta2*  *Mmp9*  *Ffar2*  *Ffar3*  *Gclc*  *Nqo1*  *Ccr2*  *Nr1h4*  *Nr0b2*  *Cyp7a1*  *Abcb11*  *Cd14*  *Lpb*  *S100a4*  *Lox*  *Il6*  *Smad2*  *Smad3*  *Smad4* | | AGCCAGAAGGGACAGTAGAA  CCATCTTTGGACCTCCCTTTAAT  GACGACTGTGTTCTCTTGTAACC  CCCAGACATCAGGGAGTAATGG  CTGGAACTCACACGACATCTT  CCACTGTATGGAGTGATCGCTG  TATGTCAGCCGTGAGAGTCCGT  GCCGAACACAAGAAGCTGGAAG  TGACCTGGCTTTAGACCCTG  CCACACCCTGTTTCGCTGTA  GGGATGAGTGTGAAGCCAGCTA  CCAAGGAGTATGCGTACCTGAAG  CACCATTCCTGCAACCTTCTGG  CCTTGGTAGAGAAGAGGCGACA  TTGAACCTCCGCAACGTGTCGT  GCAGCCGCATTTGTGATTTG  AGCTCAAGGAGCTACTGACCAG  CATCGGACTTCTTACCAAGCCG  TAGTCCTTCCTACCCCAATTTCC  TGCTGTGTTCAGGTGTTGCT  AGGAGGAGGAGAGGAGAGGA  AGTGTGCTGCTGCTGATGAT | CTCAGCCAAGCGGATGTAAA  GAAACAGACCGACATCTCTCATC  TGTTATCGTGCTCTCCTTCCCG  TCTATCGGATACTTCAGCGTCA  TCCACCTTGTTCACCTCATTT  GGGTGAAGTTCTCGTAGCAGGT  CCAGGAGTTGATGAAAGTCGGC  GGCAAATCCTGCTACGAGCACT  ACCTCTGAGCATCCATAGCC  TGCATGGCCTGGTCTAAGTG  GTGGCTGAACTTGAGGAAACGG  GCTCCAAGACTTCACACAGTGC  ATGGCATTCCCTCCAGAGCTGA  ATGGCTACCCTTTGCTTCTGCC  CGCAGGAAAAGTTGAGCGAGTG  TTCGACTCGATCTGGTTGTGG  GCTGTCCAAGTTGCTCATCACC  GGCATCAAGCAGGTCATAGTGG  TTGGTCCTTAGCCACTCCTTC  AGGTAGAGGGTGGTGTTGGT  CTGCTGCTGATGTTGTTGGT  CTGCTGCTGGTAGGTGATGT |

**Supplementary Table S2. Baseline characteristics of the three study groups**

**Supplementary Table S3. Baseline characteristics of the five study groups**
